# Ultrasonographic findings and prenatal diagnosis of Jacobsen syndrome: A case report and review of the literature

**DOI:** 10.1097/MD.0000000000018695

**Published:** 2020-01-03

**Authors:** Shuang Chen, Ruixue Wang, Xinyue Zhang, Leilei Li, Yuting Jiang, Ruizhi Liu, Hongguo Zhang

**Affiliations:** Center for Reproductive Medicine and Center for Prenatal Diagnosis, First Hospital, Jilin University, Changchun, China.

**Keywords:** fetus, Jacobsen syndrome, prenatal diagnosis, ultrasonographic findings

## Abstract

**Rationale:**

Jacobsen syndrome (JBS) is a rare chromosomal disorder with variable phenotypic expressivity, which is usually diagnosed in infancy and childhood based on clinical examination and hematological and cytogenetic findings. Prenatal diagnosis and fetal ultrasonographic findings of JBS are rare.

**Patient concerns:**

A 38-year-old, gravida 3, para 1, pregnant woman underwent clinical ultrasound examination at 22 weeks of gestation.

**Diagnoses:**

Ultrasonographic findings indicated an interventricular septal defect, the presence of septal blood flow, dilation of the left renal pelvis, and a single umbilical artery. Amniocentesis was performed to evaluate possible genetic causes of this diagnosis by cytogenetic and single nucleotide polymorphism (SNP) array analysis.

**Interventions:**

After genetic counseling and informed consent, the couple elected to terminate the pregnancy.

**Outcomes:**

Karyotype analysis showed that the fetal karyotype was 46,XX,del(11)(q23). The SNP array revealed a 6.118 Mb duplication of 11q23.2q23.3 and a 15.03 Mb deletion of 11q23.3q25.

**Lessons:**

Ultrasonographic findings of fetal JBS, including an interventricular septal defect, dilation of the left renal pelvis, and a single umbilical artery, may be associated with a 15.03 Mb deletion of 11q23.3q25. Further cases correlating phenotype and genotype are required to predict the postnatal phenotype.

## 1 Introduction

Jacobsen syndrome (JBS), also known as11q23 deletion syndrome, is a contiguous gene syndrome caused by partial deletion of the long arm of chromosome 11.^[1]^ JBS is a rare chromosomal disorder with variable phenotypic expressivity,^[2]^ and is usually diagnosed in infancy and childhood based on clinical examination and hematological and cytogenetic findings.^[3]^ Typical clinical features of JBS patients include multiple congenital abnormalities,^[4]^ dysmorphic features, congenital heart disease, intellectual disability,^[5]^ physical growth retardation, mental retardation, facial dysmorphism, visceral malformations,^[6]^ mild to moderate psychomotor retardation, trigonocephaly, cardiac defects,^[7,8]^ neonatal thrombocytopenia and persistent platelet dysfunction,^[6,9]^ dilation of the renal pelvis and seizures,^[10]^ combined immunodeficiency,^[11]^ or antibody deficiency.^[12]^ However, these characteristics exist variably between patients.^[8]^ This suggests that the phenotypes caused by different 11q deletions are inconsistent.

Since Dr Jacobsen's first report, more than 200 cases of JBS diagnosed after birth have been reported.^[5]^ However, prenatal diagnosis and fetal ultrasonographic findings are rare. We report a case of prenatal diagnosis of JBS, and describe the ultrasound findings and genetic results of a de novo duplication of chromosome 11q23.2q23.3 and deletion of chromosome 11q23.3q25. Meanwhile, this study also reviews the relationship between different 11q deletions and prenatal ultrasound findings as described in the literature.

## 2 Methods

This study was approved by the Ethics Committee of the First Hospital, Jilin University (No. 2018-383). Patient has provided informed consent for publication of the case.

### 2.1 Cytogenetic analysis

Fetal cells were obtained through amniocentesis after obtaining written informed consent. Then, amniocytes were collected by centrifugation, inoculated in flasks according to laboratory standards, and cultured in carbon dioxide incubators for 10 days. Chromosome analysis using G-band staining was performed as in our previous study.^[13]^ The karyotype was described according to the International System for Human Cytogenetic Nomenclature (ISCN 2013).^[14]^ Twenty metaphases were analyzed.

### 2.2 SNP array analysis

Genomic DNA was extracted from 10 mL of uncultured amniocytes using a QIAamp DNA Mini kit (Qiagen, Hilden, Germany) according to the manufacturer's instructions. SNP array analysis was performed using the Human CytoScan 750K BeadChip (Affymetrix, San Diego, CA). Image data were analyzed using Chromosome Analysis Suite v3.3 software. The final results were analyzed using the Database of Chromosomal Imbalance and Phenotype in Humans using Ensembl Resources (DECIPHER), the Database of Genomic Variants (DGV), OMIM, and NCBI.

## 3 Case presentation

**Figure 1:**
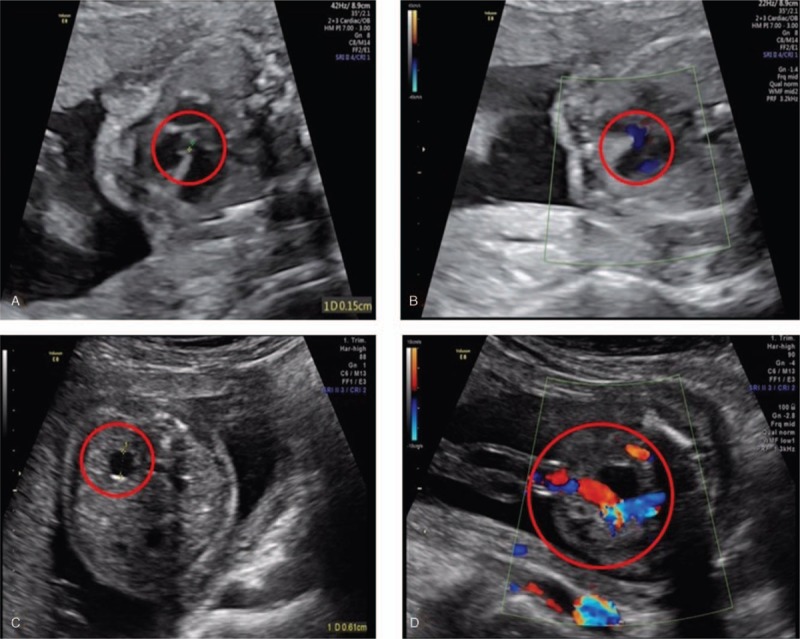
Prenatal ultrasound at 22 weeks of gestation showing: A: Interventricular septal defect; B: Presence of septal blood flow; C: Left renal pelvis widening; D: Single umbilical artery.

A 38-year-old, gravida 3, para 1, pregnant woman underwent clinical ultrasound examination at 22 weeks of gestation. Ultrasonographic findings indicated abnormalities of the single live fetus, including a single ventricle in the intracalvarium, the skull ring was completed, the width of the right ventricle was 0.89 cm and the width of the left ventricle was 0.82 cm, the biparietal diameter was 5.3 cm, the head circumference was 19.8 cm, the abdominal circumference was 16.6 cm, the femur length was 3.8 cm, and the amniotic fluid index was 13.3 cm. The main abnormal manifestations on the ultrasound images included an interventricular septal defect, the presence of septal blood flow, dilation of the left renal pelvis, and a single umbilical artery (Fig. 1). After genetic counseling, the woman was offered amniocentesis for cytogenetic and single nucleotide polymorphism (SNP) array analysis at 23 weeks of gestation because of advanced age in combination with these abnormal ultrasound indicators.

**Figure 2:**
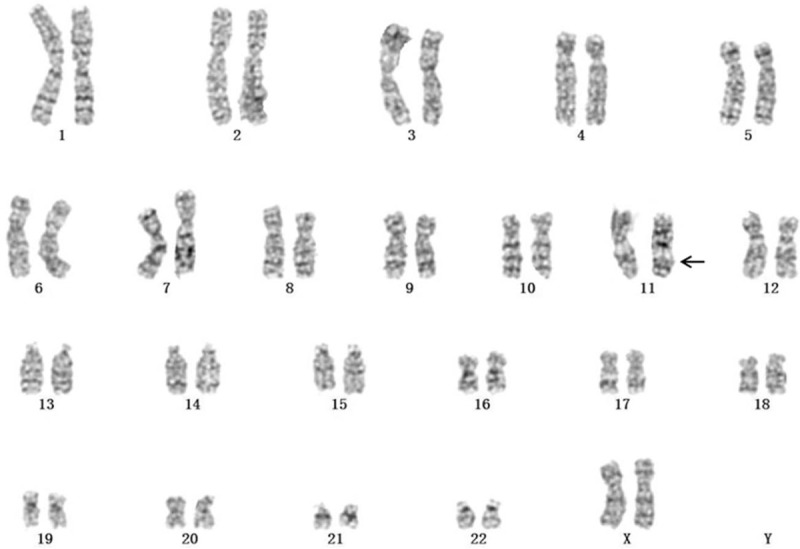
Karyotype of the fetus identified by GTG banding technique.

**Figure 3:**
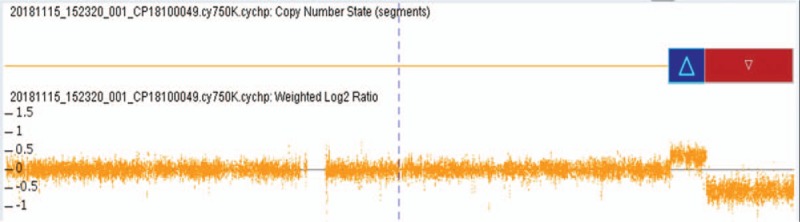
SNP array on uncultured amniocytes depicting 11q23.2q23.3 duplication and 11q23.3q25 deletion.

Karyotype analysis showed that the fetal karyotype was 46,XX,del(11)(q23) (Fig. 2). The SNP array revealed a 6.118 Mb duplication of 11q23.2q23.3 and a 15.03 Mb deletion of 11q23.3q25 (11q23.2q23.3 [113790010–119907572] × 3, 11q23.3q25 [119907627–134937416] × 1) (Fig. 3). The couple underwent cytogenetic detection. The results were both normal.

Both the parents were nonconsanguineous and healthy, and their first child was healthy. The mother denied any exposure to smoking, alcohol, infectious diseases, irradiation, or teratogenic agents during this pregnancy. The chromosome aberrations of the fetus arose de novo. This case was diagnosed as JBS according to reports in the literature of JBS with del(11)(q23.3q25). After genetic counseling and informed consent, the couple elected to terminate the pregnancy.

## 4 Discussion

JBS has an estimated occurrence of 1 in 100,000 births, with a female to male ratio of 2:1.^[15]^ Of the cases reported, 85% arose de novo and 15% may have arisen from inheritance of an unbalanced segregation of a familial balanced translocation.^[16]^ The deletion size ranged from approximately 7 to 20 Mb, and deletion of the breakpoint at 11q23.3 was found in the majority of JBS cases.^[2,16,17]^ Previous studies have shown that the fragile site in 11q23.3 is susceptible to chromosome deletion in vivo.^[18]^ The present case was a female fetus with a 15.03 Mb deletion of 11q23.3q25 and a 6.118 Mb duplication of 11q23.2q23.3.

For child patients who survive the neonatal period, more attention has been paid to the clinical phenotypes and the genes involved in the deleted regions.^[1,5,19]^ Patients with JBS show a wide spectrum of clinical phenotypes, and patients with the more obvious clinical features are diagnosed by the age of one.^[16]^ Previous studies have shown that 97% of JBS patients have mild to severe mental retardation; congenital heart malformations are observed in 56% of cases.^[16,20]^ In addition, most patients with JBS are diagnosed with either thrombocytopenia or pancytopenia. More clinical manifestations include facial dysmorphism, intellectual disability, immunodeficiency, and autism spectrum disorder.^[15]^ However, the most severe phenotypes of the patients with deletions vary between patients.

The present case reports prenatal diagnosis and ultrasound findings of a JBS case. Meanwhile, from a review of the literature, ultrasonographic findings of JBS were collected and are summarized in Table 1. The ultrasound findings of the present study showed an interventricular septal defect and the presence of septal blood flow. Foley et al^[21]^ reported fetal JBS showing hypoplastic left heart syndrome. Ye et al^[22]^ found by animal model studies that deletion of *ETS-1* related to the JBS critical region can cause ventricular septal defects. These results are consistent with the high incidence of congenital heart malformation found after birth. Here, the fetus was found to have dilation of the left renal pelvis, consistent with a report by Wax et al,^[23]^ and a single umbilical artery, consistent with a report by Chen et al.^[15]^ The ultrasound findings in the published literature vary between patients. Hence, the relationship between genotype and phenotype still needs to be studied further in more cases.

**Table 1:**
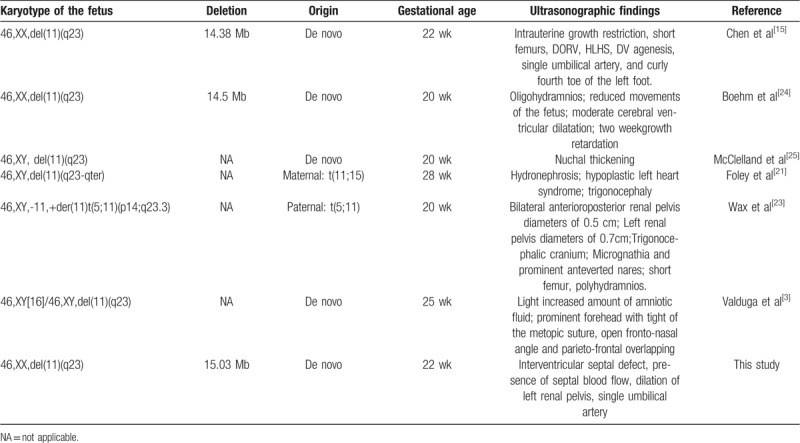
Prenatal diagnosis and ultrasonographic findings of Jacobsen syndrome in published literature.

A limitation of this study is that fetal autopsy was not performed because the couple refused consent, and so we were unable to confirm the ultrasound findings.

We present ultrasonographic findings of JBS in a fetus with an interventricular septal defect, dilation of the left renal pelvis, and a single umbilical artery. These abnormalities were associated with a 15.03 Mb deletion of 11q23.3q25. More cases correlating phenotype and genotype are required to predict the postnatal phenotype.

## Acknowledgments

We thank Catherine Perfect, MA (Cantab), from Liwen Bianji, Edanz Editing China (www.liwenbianji.cn/ac), for editing the English text of a draft of this manuscript.

## Author contributions

**Funding acquisition:** Ruizhi Liu.

**Investigation:** Ruixue Wang, Xinyue Zhang.

**Methodology:** Leilei Li, Yuting Jiang.

**Writing – original draft:** Shuang Chen.

**Writing – review & editing:** Ruizhi Liu, Hongguo Zhang.

Hongguo Zhang orcid: 0000-0001-8953-863X.
